# Prevalence of acquired drug resistance mutations in antiretroviral- experiencing subjects from 2012 to 2017 in Hunan Province of central South China

**DOI:** 10.1186/s12985-020-01311-3

**Published:** 2020-03-17

**Authors:** Xiaobai Zou, Jianmei He, Jun Zheng, Roberta Malmgren, Weisi Li, Xiuqing Wei, Guoqiang Zhang, Xi Chen

**Affiliations:** 1 Hunan Provincial Center for Disease Control and Prevention, No.450 Section 1 Furongzhong middle Road, Kaifu District, Changsha, 410005 Hunan Province China; 2 University of California at Los Angeles, 405 Hilgard Avenue, Los Angeles, CA 90095 USA; 3 Hunan Science and Technology Information Institute, No.59 Bayi Road Furong District, Changsha, 410001 Hunan China

**Keywords:** HIV, Acquired drug resistance, China, Mutation

## Abstract

**Background:**

There are few data on the prevalence of acquired drug resistance mutations (ADRs) in Hunan Province, China, that could affect the effectiveness of antiretroviral therapy (ART).

**Objectives:**

The main objectives of this study were to determine the prevalence of acquired drug resistance (ADR) the epidemic characteristics of HIV-1-resistant strains among ART-failed HIV patients in Hunan Province, China.

**Methods:**

ART-experienced and virus suppression failure subjects in Hunan between 2012 and 2017 were evaluated by genotyping analysis and mutations were scored using the HIVdb.stanford.edu algorithm to infer drug susceptibility.

**Results:**

The prevalence of HIV-1 ADR were 2.76, 2.30, 2.98, 2.62, 2.23and 2.17%, respectively, from 2012 to 2017. Overall 2295 sequences were completed from 2932 ART-failure patients, and 914 of these sequences were found to have drug resistance mutation. The most common subtype was AE (64.14%), followed by BC (17.91%) and B (11.50%). Among those 914 patients with drug resistance mutations,93.11% had NNRTI-associated drug resistance mutations, 74.40% had NRTI drug resistance mutations (DRMs) and 6.89% had PI DRMs. Dual-class mutations were observed in 591 (64.66%) cases, and triple-class mutations were observed in 43 (4.70%) cases. M184V (62.04%), K103N (41.90%) and I54L (3.83%) were the most common observed mutations, respectively, in NRTI-, NNRTI- and PI-associated drug resistance. 93.76% subjects who had DRMs received the ART first-line regimens. CD4 count, symptoms in the past 3 months, and ART adherence were found to be associated with HIV-1 DR.

**Conclusions:**

This study showed that although the prevalence of HIV-acquired resistance in Hunan Province is at a low-level, the long-term and continuous surveillance of HIV ADR in antiretroviral drugs (ARVs) patients is necessary.

## Background

HIV has been a leading cause of infectious diseases and death globally over the last three decades. Since highly active antiretroviral therapy (HAART) became available, the HIV-infected patients’ life expectancy and health outcomes have been drastic improved. However, the widespread use of HARRT has led to drug resistance (DR) to ARVs, which undermines their effectiveness [1, 2]. Drug resistance can be categorized into ADR mutations in treatment-experienced patients and transmitted drug resistance (TDR) mutations in treatment-naïve individuals, both of which could cause the failure of the antiretroviral therapy (ART). Ignoring TDR can result in treatment failure of antiretroviral regimens, and ADR is often associated with virological failure (VF) and can increase the burden of treatment [3, 4]. While TDR has been well documented, fewer studies report on the rates of ADR. A systematic review from resource limited settings reports on rates of ADR steadily increased with time on ARV [5].. M184V is the most commonly occurring NRTI ADR mutation. In vitro, it causes high-level resistance to lamivudine (3TC). The next most common ADR mutation is the K103N mutation (associated with non-nucleoside reverse transcriptase inhibitors; NNRTIs) which cause high-level resistance to nevirapine (NVP) and variable resistance to efavirenz (EFV) [6].

Since the first case of HIV infection was found in 1992, there were 29,002 people living with HIV (PLWH) to the end of 2017 in Hunan province, which is in south central China, and has a population of 68 million. More than 25,530 HIV/AIDS patients had received free highly active antiretroviral therapy (HAART) and were still in treatment which is supported by the government by the end of 2017. Although stavudine (D4T) and zidovudine (AZT) are not recommended as first-line therapy in the US and Europe, in much of Asia and other resource-limited settings, the most common first-line regimens for HIV-infected patients still contain either D4T or AZT, which are less costly than tenofovir (TDF) [7–9]. The first line regimen of HAART given to patients in Hunan Province China is a triple therapy choosing from d4T, AZT, TDF, 3TC, Efavirenz (EFV) and nevirapine (NVP), all these generic drugs produced in China. Lopinavir/ritonavir (LPV/r), produce outside China, is the second-line drug. A recent Hunan Province molecular epidemiology survey showed that 4 HIV-1 subtypes in Hunan province reported were CRF_01AE, CRF07_BC, B and C. CRF_01AE was the dominant subtype strain (more than 70%) [10–12].

The emerging ADR and subsequent treatment failure pose a major concern for HIV ART in resource-limited settings where treatment options are limited. The objective of this study is to determine the prevalence and DRMs of HIV-1 virologic failure and acquired drug resistance among ART-experienced adults in Hunan Province, China.

## Methods

### Ethical statement

The research protocol, approved by the relevant institutional review boards or independent ethics committees, was conducted in accordance with standards for the protection of patient safety and welfare and in compliance with Good Clinical Practices and the principles of the Declaration of Helsinki and its amendments. According to “National Free AIDS ARV guidelines” (China), all participants who receive treatment from AIDS ARV supported by the Chinese government must have viral load testing and genotypic testing, which will be used for this study. Verbal informed consent is given to the doctors at the clinical sites who are responsible for the AIDS patients, and the patients’ names were kept on a list with the doctors’ signature. The study and the verbal consent procedure were approved by the Ethical Committee of Hunan Provincial Center for Disease Control and Prevention (ethical approval number: 201801).

### Subjects

The Chinese national free ARV treatment policy guidelines recommend that patients on ART should have an HIV viral load test at least once a year [10–12]. If the viral load is > 1000 copies/ml, a HIV drug resistance genotypic test (Sanger Sequencing based) should be performed. All the patients seen between 2012 to 2017 who had taken ART more than 6 months and had a test for virus load (VL) by the Hunan CDC HIV Laboratory were enrolled in this study. Primary inclusion criteria: 1) HIV-infected; 2) on ART more than 6 months; 3) a minimum of 18 years of age; 4) a plasma HIV RNA > 1000 copies/mL with any CD4+ lymphocyte count. The data were extracted from the Chinese National HIV DR Surveillance database, Questionnaire data from the Chinese HIVDR database on age, ethnicity, gender, profession, education, marital status, route of HIV transmission, initial ART regimen and missed doses in the past month were collected by trained interviewers [13]. The viral load testing and drug resistance testing were done by the Hunan CDC HIV Laboratory.

### Laboratory tests

#### CD4^+^ T cell abs count and viral load testing

CD4^+^ T cell abs counts were determined with a FACS Calibur (Becton Dickinson, Mountain View, California, USA) in freshly collected whole blood (within 24 h). Plasma HIV-1-RNA viral loads were determined using the COBAS TaqMan HIV-1 test, version 2.0 (CAP/CTM v2.0) with a lower limit of detection of 40 RNA copies/ml and Abbott m2000 Diagnostics Systems with a lower of detection of 150 RNA copies/ml.

#### RNA extraction, amplification and sanger sequencing

HIV RNA was then extracted from 140 ul of plasma using the QIAmp Viral RNA mini kit (Qiagen, Germany) according to the manufacturer’s protocol. Approximately 1247 bp pol fragments (HXB2 positions 2057–3304) were amplified by nested reverse transcriptase polymerase chain reaction (nested RT-PCR). The 1st round PCR using one-step RT PCR kits (Promega, USA) with primers: F1a (5′- TGAARGAITGYACTGARAGRCAGGCTAAT − 3′), F1b(5′- ACTGARAGRCAGGCTAATTTTTTAG − 3′) and RT-R1(5′-ATCCCTGCATAAATCTGACTTGC − 3′) and the cycling conditions were as follows: incubation at 50 °C for 45 min, 94 °C for 2 min, then 35 cycles of 94 °C for 15 s, 50 °C for 20 s, and 72 °C for 2 min 30 s, the 72 °C for 10 min. The 2 ^st^ round was implemented using Taq PCR Mastermix (Tiangen, Beijing, China) with primers: PRT-F2(5′- CTTTARCTTCCCTCARATCACTCT − 3′) and RT-R2(5′-CTTCTGTATGTCATTGAC AGTCC − 3′) and the condition were: 94 °C for 4 min, then 30 cycles of 94 °C for 15 s,55 °C for 20 s, and 72 °C for 2 min and then 72 °C for 10 min. The PCR products were purified and sequenced by the North Genomics Research Center (Sinogenomax Ltd., Beijing).

#### Sequence and mutation analysis

The results from sequencing were aligned and assembled manually by Bio Eidt (7.0.2) and ContigExpress Project, a component of the Vector NTI Suite 6 software, Major drug-resistance mutations were defined according to the IAS-USA 2015 list [14]. The subtype analysis and the level of clinically relevant loss of antiviral activity for each drug was determined using the Stanford HIVdb algorithm (http://hivdb.stanford.edu), with a resistance score of ≥15 for any drug [14, 15]. Statistical analyses were conducted using STATA 15.0 (STATA Corp, Texas, USA). Findings with ***P*** < 0.05 were used to determine statistical significance.

## Results

### Demographics characteristics

For the years 2012 to 2017, there were 1520, 3650, 6749, 8215 and 9380 patients respectively who received ART more than 6 months and who had done the VL testing. The proportion of patients with ART failure (VL >1000copies/ml) was 8.55% (130/1520), 8.30% (303/3650), 8.67% (585/6749), 8.23% (675/8215), 7.49% (703/9380) and 7.12% (536/7524).

Most of the subjects were male (71.59%, 2099/2932). The median age was 44. (IQR: 33–52), and the age distribution was mainly in adults aged 30–44 years old, but the percentage of the older population (> 60) has increased year by year (from 12.31 to 25.19%). Half of the study population were married or cohabiting (52.49%,1539/ 2932). The most common route of infection was heterosexual (73.91%, 2167/2932), the proportion of PWID (persons who inject drugs) showed a declining pattern each year from 2012 to 2017(from 19.23% to 4.66 and MSM increased year by year, (from 2.31 to 14.93%).The median virus load of participants was 18,882 copies/ml (IQR 5215–63,000), and the median CD4 was 189 cells/ml (IQR:81–277). (Table 1.)

**Table 1:** Demographic Characteristics of the ART-Failure patients in Hunan Province from 2012 to 2017

	2012(*n* = 130)	2013(*n* = 303)	2014(*n* = 585)	2015(*n* = 675)	2016(*n* = 703)	2017(*n* = 536)	Total(*n* = 2932)
**Gender**							
Male	83	208	401	492	520	395	2099
Female	47	95	184	183	183	141	833
**Age**							
< 14	3	3	14	5	6	5	36
15~29	16	37	85	111	125	96	470
30~44	60	134	229	210	192	171	996
45~59	35	80	141	196	208	129	789
≥ 60	16	49	116	153	172	135	641
Median and IQR	41 (33–49)	43 (32–54)	42 (33–52)	45 (34–58)	46 (33–59)	44 (31–56)	44 (33–52)
**Route of Infection**							
BLOOD transmission	6	10	7	5	5	8	41
PWID	25	50	67	69	47	25	283
MSM	3	19	45	58	76	80	281
Heterosexual	88	214	431	506	537	391	2167
Vertical transmission	3	3	14	5	6	8	39
Unkown	5	7	21	32	32	24	121
**Marital Status**							
Single	25	70	136	146	189	159	725
Married/cohabitation	76	156	319	376	349	263	1539
Divorced/separated	14	41	86	101	109	75	426
Widowed	15	36	39	49	52	36	227
Unknown	0	0	5	3	5	3	16
**HIV RNA levels (Median, IQR)**	14,950 (5090–63,500)	26,405 (6813–74,022)	29,361 (6791–79,763)	18,344.5 (5162–68,838)	14,631 (4315–51,518)	15,717 (4760–51,048)	18,882 (5215–63,000)
**CD4 levels (Median, IQR)**	167 (74–247)	182.5 (89–257)	174.5 (76–255)	188.5 (81–275)	200.5 (81–287)	208 (89.5–306.5)	189 (81–277)

HIV RNA levels expressed in copies/ml, CD4 levels expressed in cells/mm^3^

*PWID* Persons who inject drugs, *MSM* Man have sex with man, *IQR* Interquartile range

### Prevalence of HIV drug resistance mutation

Among the 2932 ART failures, 2295 *pol* area complete sequences (1200 bp) were obtained by Sanger sequencing. The primary subtype was AE (64.14%, 1472/2295), followed by BC (17.91%, 411/2295) and B (11.50%, 264/2295) (Table 2). Although the AE subtype was the most common strain in most cities in Hunan Province, in Zhangjiajie City subtype B was the main strain.

**Table 2:** The subtype distribution of the ART failures patients from 2012 to 2017. and the different drug resistance classes (single/double/ Triple drug resistance) in these patients

Year	2012	2013	2014	2015	2016	2017	total
**ART Failure**	130	303	585	675	703	536	2932
**Obtained sequence**	112	239	508	511	528	397	2295
**Subtype**							
*AE*	75 (66.96%)	163 (68.20%)	355 (69.88%)	365 (71.43%)	294 (55.68%)	220 (55.42%)	1472 (64.14%)
*BC*	11 (9.82%)	26 (10.88%)	80 (15.75%)	106 (20.74%)	115 (21.78%)	73 (18.39%)	411 (17.91%)
*B*	21 (18.75)	37 (15.48%)	52 (10.24%)	30 (5.87%)	66 (12.50%)	58 (14.61%)	264 (11.50%)
*C*	4 (3.57)	13 (5.44%)	20 (3.94%)	9 (1.76%)	23 (4.53%)	12 (3.02%)	81 (3.53%)
*Other*	1 (0.89%)	0	1 (0.20%)	1 (0.20%)	30 (5.68%)	34 (8.56%)	67 (2.92%)
**DRMs**	42	84	201	215	209	163	
*Only PI*	0	0	1	3	4	2	10
*Only NRTI*	1	1	13	6	21	7	49
*Only NNRTI*	6	23	57	56	50	29	221
*PI and NRTI*	0	0	0	1	1	2	4
*PI and NNRTI*	0	0	0	0	3	1	4
*NRTI and NNRTI*	35	60	129	144	121	94	583
*PI and NRTI and NNRTI*	0	0	1	5	9	28	43

Note: *ART* Antiretroviral therapy

*DRMs* Drug resistance mutations

*PI* Protease inhibitor

*NRTI* Nucleoside reverse transcriptase inhibitors

*NNRTI* Non-nucleoside reverse transcriptase inhibitors

Nine hundred and fourteen of 2295(39.83%) had at least one primary drug resistance mutation (DRM) according to the Stanford HIVdb algorithm SDRM list of mutations [16]. The annual acquired drug resistance (ADR) rates for 2012 to 2017 were 2.76%(42/1520), 2.30%(84/3650), 2.98%(201/6749), 2.62%(215/8215), 2.23%(209/9380), and 2.17%(163/7524). Ninety-three percent (851/914) of cases had NNRTI-associated drug resistance mutations; 680 cases (74.40%) had mutations associated with NRTI resistance, and 63 had PI DRMs (6.89%). Dual-class mutations were observed in 591 (64.66%) cases: 583 NRTI + NNRTI, four NRTI + PI and four NNRTI + PI. Triple-class mutations were observed in 43 (4.70%) sequences (Table 2). The most common primary NRTI mutations detected included M184V (62.04%), K65R (20.24%), K70R (15.01%), T215D/S (9.41%), Y115F (7.22%) and M41L (6.35%). The most frequent mutations to NNRTIs were K103N (41.90%), Y181C (28.12%), G190A (26.48%), K101E (11.71%), V106M/A (10.94%), V108I (7.44%), 221Y (7.22%) and Y188H (5.91%).The proportion of PI-associated DRMs showed a tendency to increase from 2012 to 2017(0, 0, 0.22, 0.98, 1.86 and 3.81%). The major mutations were I54L (3.83%), V82L (2.84%) and M46I/L (1.53%). All of these can lead to LPV/r resistance (Table 3). The NNRTI (t)-associated mutation G190A emerged in 92.24% (202/219) of the subtype CRF01AE sequences and in 7.76% of other subtypes (*p* < 0.05). Y181C also emerged frequently in subtype CRF01AE sequences (71.60% vs 28.40% for other subtypes, p < 0.05).

**Table 3:** The major acquired drug resistance mutations found in this study. The drug resistance mutations were identified using the Stanford University HIVdb algorithm

	2012 (*n* = 42)	2013 (*n* = 84)	2014 (*n* = 201)	2015 (*n* = 215)	2016 (*n* = 209)	2017 (*n* = 163)	Total (*n* = 914)
**PI**							
M46I/L	0	4	2	4	5	4	14 (1.53%)
I84V	0	0	1	1	1	1	4 (0.44%)
L76V	0	0	1	3	5	2	11 (1.20%)
L90M	0	0	1	1	1	0	3 (0.33%)
F53L	0	0	0	1	0	0	1 (0.11%)
I50IV	0	0	0	2	0	3	5 (0.55%)
I54L	0	0	0	3	7	25	35 (3.83%)
V82L	0	0	0	3	6	17	26 (2.84%)
**NRTI**							
M184V/I	36	53	124	129	118	108	567 (62.04%)
T69S/D/N	1	3	4	15	9	4	35 (3.83%)
T215D/S	9	16	20	17	18	7	86 (9.41%)
M41L	2	7	10	6	18	16	58 (6.35%)
K70Q/R	10	12	21	34	36	26	138 (15.10%)
K65R	3	6	33	50	43	51	185 (20.24%)
L210W	4	6	6	3	9	5	32 (3.50%)
K70R/E	10	13	20	34	36	26	138 (15.10%)
Y115F	3	1	11	17	22	13	66 (7.22%)
L74I	1	2	11	15	20	10	58 (6.35%)
**NNRTI**							
K103N/S	14	42	85	91	75	76	383 (41.90%)
K101E	9	11	22	26	22	17	107 (11.71%)
Y181C	11	26	69	64	49	39	257 (28.12%)
221Y	6	12	17	9	16	6	66 (7.22%)
Y188H	2	7	14	13	8	10	54 (5.91%)
G190A	14	23	53	61	55	37	242 (26.48%)
P225H	2	5	15	12	8	6	48 (5.25%)
A98	2	3	12	14	9	10	50 (5.47%)
V108I	3	10	17	12	17	9	68 (7.44%)
V106M/A	2	2	17	37	25	17	100 (10.94%)
L100I	0	0	4	5	9	3	31 (3.39%)
M230L	0	1	6	11	6	7	31 (3.39%)

Note: HIV drug resistance mutations were identified using the Stanford University HIVdb algorithm

*ART regimens*

Eight hundred and fifty-seven of 914 subjects (93.76%) who had DRMs received the ART first-line regimens that include two NRTIs and one NNRTIs. All the regimens contain 3TC, 468 patients took AZT + NVP/EFV, 102 took D4T + NVP/EFV and 287 took TDF + NVP/EFV. 6.13% of patients (56/914) received the second-line regimens which were made with LPV/r and two NRTIs /NNRTIs. Only 1 patient took the Raltegravir (RAL) + 3TC+ Abacavir (ABC). Among the 1381 no DRMs subjects, 882(63.87%, 882/1381) took the first-line regimens: 424 took AZT + NVP/EFV, 62 took D4T + NVP/EFV and 396 took TDF + NVP/EFV; 499(36.13%, 499/1381) received the second-line regimens (LPV/r or RAL or ABC + 3TC+ NVP).

### Factors associated with the detection of ADRs

Nine potential risk factors were considered in the multivariate logistic regression analysis (Table 4). Of these factors, the CD4+ T cell count, symptoms in recent three months and ART non-adherence (missed doses in the prior 7 days) were related to HIV-1 DR (*p* < 0.05). The odds of having a drug resistance mutation were 3.15 times higher for a patient who had low-level CD4 count(≤200 cells/mm^3^) than for a patient who had a high-level CD4 count(> 200 cells/mm^3^)(95% CI:2.54–3.89). The patients who had symptoms in the last 3 months had a higher risk for developing drug resistance mutation compared with those with no symptoms in the last 3 month (OR:1.48, 95%CI:1.18–1.85). There were 59.63% (545/914) DRMs detected subjects who reported missing a dose in the last 7 days, and compared to 37.29% (515/1381) no mutation detected subjects who missed a dose in the last 7 days (OR:2.94,95% CI:2.38–3.63). The patients taking regimens containing TDF or LPV/r had fewer resistance mutations compared with the patients on regimens containing AZT (*P* < 0.05). The age, gender, subtype, WHO clinical stage and routes of infection did not show significantly differences between DRMs identified and no DRM identified group (*P* > 0.05).

**Table 4:** Nine potential risk factors were considered the association with incidence of HIV-1 DRMs among participants experiencing treatment failure

Variable	Mutation identified (***n*** = 914)	No mutations identified (***n*** = 1381)	OR	P	95%CI
**Gender**					
Male	654	994	0.95	0.68	0.76–1.20
Female	260	387	1		
**Age**					
< 14	20	12	1		
15~29	146	224	0.22	0.32	0.11–4.37
30~44	314	461	0.21	0.31	0.01–4.22
45~59	255	374	0.18	0.26	0.01–3.61
≥ 60	179	310	0.20	0.30	0.01–4.06
**Regimen**					
AZT + 3TC + NVP/EFV	468	424	1		
D4T + 3TC + NVP/EFV	102	62	1.39	0.10	0.94–2.05
TDF + 3TC + NVP/EFV	287	396	0.40	0.00	0.32–0.51
Second line regimens	57	499	0.08	0.00	0.06–0.11
**Subtype**					
AE	607	866	1		
BC	126	285	0.62	0.00	0.48–0.81
B	103	160	0.80	0.16	0.59–1.09
C	36	45	1.18	0.55	0.69–2.00
Other	42	25	1.36	0.28	0.77–2.39
**CD4 abs count (cells/mm**^**3**^**)**					
≤ 200	685	705	3.16	0.00	2.55–3.91
> 200	229	676	1	1.00	
**WHO Stage**					
I	449	695	1		
II	237	358	0.9	0.41	0.70–1.16
III	129	214	0.81	0.20	0.59–1.12
IV	99	114	1.08	0.68	0.74–1.59
**Route of transmission**					
BLOOD transmission	12	18	1		
PWID	79	148	1.02	0.96	0.42–2.50
MSM	90	128	1.44	0.43	0.58–3.58
Heterosexual	676	1024	1.27	0.58	0.55–2.95
Vertical transmission	21	13	0.49	0.64	0.02–9.80
Unknown	36	50	1.32	0.58	0.50–3.49
**Recently 7 days number of doses missed**					
0	369	866	1		
≥ 1	545	515	2.94	0.00	2.38–3.63
**Symptoms in recent three months**					
No			1		
Yes			1.48	0.00	1.18–1.85

Note: *OR* Odds ratio;

*CI* Confidence interval

*P*-Values in bold are statistically significant at the 0.05 significance level

## Discussion

Our study aimed to elucidate the prevalence of HIV ADRs in ART VF subjects, since ADR is one major problem with the efficacy of ART as it affects the clinical outcomes of treatment. In this study, the annual ADR rate was lower than the threshold low-incidence rate defined by WHO of 5% [17] (from 2012 to 2017: 2.76, 2.44, 2.98, 2.68, 2.24 and 2.19%). This situation is mainly due to the expansion of National Free Antiretroviral Treatment Program (NFATP) in Hunan Province in recent years. Early antiviral treatment can control virus replication as early as possible, and reduce the possibility of drug resistance [13, 18]. The result showed that CRF01 AE is still the most dominance subtype in Hunan province (64.14%), followed by subtype BC,B and C, the finding corresponding to the last years of the Hunan Province molecular epidemiology survey (2009–2015) results [10–12, 19].. The prevalence of HIV-1 subtype B was significantly higher in Zhangjiajie City compared with other areas in Hunan province. Most of the HIV infected people were older people, who lived same area. Previous studies have had similar findings [20]. A HIV epidemic outbreak of subtype B occurred in former plasma donors (FPDs) in central China in 1990s [21, 22]. Some of the FPDs came from Zhangjiajie city, and as a result the B strains were introduced into Zhangjiajie. Due to the economic backwardness of the region, traffic congestion, limited emigration, and open sex, the B strain became a localized epidemic.

In addition to the common subtypes, other subtypes including combined subtype CRF06cpx, CRF09cpx, CRF18cpx and CRF45cpx were found in the last 2 years (2016 and 2017). This may indicate a more complicated and diverse trend of HIV-1 epidemiology emerging in Hunan province.

The most frequently identified ADR present in NRTI was M184V/I (61.27%), followed by K65R (19.96%). The elevated presence of M184V/I is expected and arises as a consequence of the use of 3TC as part of all the first-line regimens in China [23]. Although M184V/I causes high-level in vitro resistance to 3TC, M184V/I is not a contraindication to continued treatment with 3TC because it increases susceptibility to AZT, and in addition, it is associated with clinically significant reductions in HIV-1 replication [14, 24, 25]. Similar to our study finding, a high prevalence of M184 V/I mutation has been reported in Asia, Sub-Saharan Africa and Latin America, but a lower prevalence in western Europe [26–31]. This difference can be explained by the more frequent use of 3TC in low- and middle-income countries than in West European countries. K65R is the signature mutation associated with TDF resistance [32, 33]. This study showed that K65R codon mutation had a significant increase from 2014 (71.4, 6.74, 16.42, 22.73%, 20.48 and 30.91%, respectively, from 2012 to 2017), due to the widespread use of TDF replacing AZT and D4T as a part of the first-line treatment from 2014 in China following WHO recommendations [34].

NNRTIs are notorious for rapidly triggering the emergence of drug-resistant HIV-1 variants. One NNRTI drug mutation can lead to multiple and high-level NNRTI-associated drug resistance [35]. The prevalence of NNRTIs DRMs (91.91%) was higher than NRTIs (74.00%) and PIs (7.44%) in this study. This is due to the fact that NNRTIs have a low genetic barrier to resistance and a single major mutation was often leads to multiple and high-level resistance to NNRTIs drugs [30, 35, 36], which is why the second-line therapy did not include NNRTIs. K103N/S (41.32%) was the most frequently observed resistance mutation in NNRTIs, followed by Y181C(27.83%) and G190A(26.21%), This is a consequence of the frequent use of NNRTI-based (EFV/NVP) first-line therapy for more than a decade in China, and these results are similar to the data from low- and middle-income countries [27, 37–39]. K103N/S was strongly associated with failure against NVP and EVP. Y181C reduces susceptibility to NVP by > 50-fold and to EFV by 2-fold. G190A caused high-level resistance to NVP and intermediate resistance to EFV and could result in attenuation of the resistance that occurs with K103N alone, and G190A had a higher frequency (83.47%) of drug resistance in the HIV-1 CRF 01AE subtype in this study.

The overall rate of resistance to PIs was low (7.44%) in our study. This might be explained by the low PI coverage in China as a second-line regimen; and since PIs have a much higher barrier to resistance, patients receiving PI-based regimens are less likely to develop resistance. Our study results showed increasing trends in PIs mutations year by year from 2012 to 2017(from 0 to 8.31%), associated with the increasing use of second-line regimens in recent years. Therefore, it is advisable to include surveillance of PIs mutation in the coming years.

Our study results revealed that the patients’ low-level CD4 count (≤200 cells/mm^3)^, clinical symptoms and bad medication compliance (missing drugs) could more easily lead to HIV-1 drug- resistance mutation among participants experiencing treatment failure. These phenomena are a reminder that monitoring resistant strains using CD4 counts and clinical symptoms in the course of ART as well as evaluating patients’ therapy compliance are very important. TDF has been commonly used as the first-line regimen since 2014, and LPV/r has always been used as the second-line regimen. The regimens containing TDF or LPV/r resulted in less drug resistance compared with AZT-contained ARVs because they were not used as much as AZT in Hunan Province. But this study result showed that the PI related mutation had an tendency to increase year by year from 2012 to 2017. This means that in the future we should pay more attention to second-line regimens drug resistance surveillance.

Several limitations of this research should be noted. First, in this study we focused on PI- and RT-associated mutations as the most commonly used ARV classes in Hunan province up to 2017. However, some patients have used integrase inhibitor drugs at their own expense in recent years, and the picture of integrase-associated DRMs remains blank and warrants further study. A second deficiency in our research is that a moderate proportion of subjects did not return for cd4 count or viral load monitoring, and the surveillance frequency of HIV virus load (VL) was relatively low (free VL testing once a year) due to local government policy, all of which may have reduced our power to detect associations between ADR and virologic outcome.

## Conclusions

In contrast with our previous research, we focused on HIV ADR in Hunan Province during the last 6 years of this study. Although free antiviral therapy has been widely practiced in Hunan Province for more than 15 years, the prevalence of HIV-acquired resistance in Hunan Province is at a low-level. However, the resistant strains are common among those with viral suppression failure. Therefore, long-term and continuous surveillance of HIV ADR in ARV patients is necessary.

## Data Availability

The datasets during and/or analyzed during the current study available from the corresponding author on reasonable request.

## Abbreviations

3TC: Lamivudine
ABC: Abacavir
ADR: Acquired drug resistance
ADR: Acquired drug resistance mutations
ART: Antiretroviral therapy
AZT: Zidovudine
D4T: Stavudine
DR: Drug resistance
DRMs: Drug resistance mutations
EFV: Efavirenz
HAART: Highly active antiretroviral therapy
LPV/r: Lopinavir/ritonovir
NVP: Nevirapine
NVP: Nevirapine
PLWH: People living with HIV
RAL: Raltegravir
TDF: Tenofovir
TDR: Transmitted drug resistance
VF: Virological failure
VL: Virus load
